# A Short Bout of Exercise Prior to Stroke Improves Functional Outcomes by Enhancing Angiogenesis

**DOI:** 10.1007/s12017-019-08533-x

**Published:** 2019-04-02

**Authors:** Stefano Pianta, Jea Young Lee, Julian P. Tuazon, Vanessa Castelli, Leigh Monica Mantohac, Naoki Tajiri, Cesar V. Borlongan

**Affiliations:** 1 Center of Excellence for Aging and Brain Repair, University of South Florida College of Medicine, 12901 Bruce B Downs Blvd, Tampa, FL 33612 USA; 2 Department of Neurophysiology & Brain Science, Graduate School of Medical Sciences & Medical School, Nagoya City University, Nagoya, 467-8601 Japan; 3 Department of Neurosurgery and Brain Repair, University of South Florida Morsani College of Medicine, 12901 Bruce B Downs Blvd, Tampa, FL 33612 USA

**Keywords:** Ischemic stroke, Physical activity, Vasculature, Stem cells, Neuroprotection

## Abstract

Stroke remains a significant unmet clinical need with limited therapeutic options. The peculiar feature of ischemic stroke is the interruption in brain circulation, resulting in a cascade of detrimental cerebrovasculature alterations. Treatment strategies designed to maintain potency of the cerebrovasculature may protect against stroke. The present study assessed the effects of short bouts of exercise prior to stroke induction and characterized cerebral blood flow and motor functions in vivo. Adult Sprague–Dawley rats were exposed to a single short bout of exercise (30-min or 60-min forced running wheel) then subjected to transient middle cerebral artery occlusion (MCAO). Non-exercise stroke rats served as controls while non-stroke rats represented shams. Cerebral blood flow (CBF) was evaluated by laser Doppler at baseline (prior to MCAO), during MCAO, and during reperfusion. Behavioral tests using the elevated body swing test was conducted at baseline, day 0 (day of stroke), and at days 1 and 3 after stroke. Animals that received exercise displayed typical alterations in CBF after stroke, but exhibited improved motor performance compared to non-exercise rats. Exercised stroke rats showed a reduction in infarct size and an increased number of surviving cells in the peri-infarct area, with a trend towards prolonged duration of the exercise. Immunofluorescence staining and Western blot analysis of the peri-infarct area revealed increased levels of endothelial markers/angiogenesis markers, VEGF, VEGFR-2, and Ang-2, and endothelial progenitor cell marker CD34+ in exercise groups compared with the controls. These results demonstrated that prophylactic exercise affords neuroprotection possibly by improving cerebrovascular potency.

## Introduction

Stroke represents one of the most detrimental diseases worldwide (Go et al. 2014; Meschia and Brott 2018). Despite the scientific advances, paucity in achieving significant clinical positive outcomes still plagues stroke. Stroke has a high financial burden in the US, costing an estimated $34 billion each year (Benjamin et al. 2017). Moreover, the only Food and Drug Administration (FDA)-approved drug, tissue plasminogen activator (tPA), has a limited time window. In fact, tPA administration outside this window induces adverse effects, such as hemorrhagic transformation (Go et al. 2014). Additionally, tPA only benefits approximately 5% of ischemic stroke patients when treated within the 4.5 h treatment window (Emberson et al. 2014). Aside from first-line thrombolytic tPA therapy, mechanical thrombectomy has also been shown to be beneficial in the acute ischemic stroke setting (Primiani et al. 2019; Mokin et al. 2013). However, mechanical thrombectomy for clinical use encounters some issues, including a small number of suitable patients for thrombectomy in the 6–24-h time window (Campbell et al. 2015; Furlan 2015; Cohen at al. 2015); and the expertise and resources for this therapy appear restricted (Josephson and Kamel 2018; Sheth et al. 2015). As the repertoire of effective treatments is limited, preclinical and clinical studies for novel stroke treatments are warranted.

Physical activity or exercise may help promote and maintain memory and cognitive performance in healthy, elderly individuals (Vance et al. 2016; Gheysen et al. 2018). Aerobic physical activity plays a crucial role in promoting cardiovascular fitness, aerobic fitness, quality of life, cognitive performance, walking speed and endurance, balance, mobility, and other health outcomes among post-stroke patients (Oberlin et al. 2017; Billinger et al. 2014). The American Heart Association recommends regular aerobic exercise as part of stroke prevention and treatment (Winstein et al. 2016).

Physical activity’s neuroprotective mechanisms include different neuroprotective and regenerative processes, among which include neurogenesis (Brandt et al. 2010), neuronal apoptosis inhibition (Liebelt et al. 2010), cerebral inflammation reduction (Ding et al. 2005; Barrientos et al. 2011), and blood brain barrier (BBB) and neurovascular unit maintenance (Ding et al. 2006). The positive effects of exercise on stroke injuries in animal models have been previously reviewed, particularly in middle cerebral artery occlusion (MCAO) models (Zhang et al. 2011). Of note, vascularization is important to stroke recovery (Ishiwaka et al. 2013; Shinozuka et al. 2013), and physical activity helps to maintain cerebrovascular activity and integrity, and promote cerebral blood flow during reperfusion (Zwagerman et al. 2010), probably through the expression of endothelin-1 (ET-1) (Zhang et al. 2014). Intense exercises (treadmill running 5 days/week for 8–19 weeks) have been demonstrated to reduce infarct volume post-MCAO and improve NOS-dependent vascular reactivity of cerebral arterioles (Arrick et al. 2014). Moreover, exercise promotes neuronal survival and improves vascular activity and cerebral blood flow post-induced stroke in animal models, increasing vascular endothelial growth factor (VEGF) and insulin-like growth factor (IGF), which are pivotal in cerebral vasculature angiogenesis (Dornbos et al. 2013; Carro et al. 2000; Cotman et al. 2007; Tang et al. 2010). Furthermore, running activities post-MCAO in mice increase hippocampal neurogenesis and improve behavioral test scores, elevating functional outcomes in post-stroke recovery (Woitke et al. 2017). Altogether, physical activity’s positive effect in stroke is well documented in preclinical studies (Zhang et al. 2013; Pan et al. 2017; Stradecki-Cohan et al. 2017), as well as in clinical trials and meta-reviews (Belfiore et al. 2018; Oberlin et al. 2017; Robertson et al. 2017), and continues to be recommended (Billinger et al. 2014).

A few reports, however, contradict the functional benefits of exercise in stroke, highlighting the need to optimize the regimen intensity, initiation time, and exercise style, as well as elucidate the underlying mechanism. For instance, physical activity at 24 h post-focal cerebral ischemia increases cortical tissue injury (Xing et al. 2018). Very early timing of exercise post-stroke elevates pro-inflammatory cytokines and oxidative stress pathways, aggravating tissue lesions and reperfusion damage (Li et al. 2017). Early stage exercise-induced cell apoptosis leads to nitric oxide synthase (NOX) activation and hyper-glycolysis (Shen et al. 2016). Very early physical activities (within 6 h) trigger pro-apoptotic factor expression and thus, cell apoptosis (Li et al. 2017). Moreover, early stage exercise exacerbates cerebral oxygen metabolism, altering oxidative phosphorylation and resulting in a reduction of ATP generation (Hasan et al. 2016).

In a few clinical studies, weak to moderate exercise prior to stroke has attenuated stroke severity (Deplanque et al. 2006, 2012; Krarup et al. 2008). Exercise may be considered a mild stressor (Morton et al. 2009; Arumugam et al. 2006) and thus follows the prototypical preconditioning stimulus, exerting neuroprotection and improving cognitive function (Mattson 2012; Voss et al. 2013). The peculiar feature of ischemic stroke is the interruption in brain circulation, triggering a cascade of deleterious cerebrovasculature alterations (Lo et al. 2003; Cai et al. 2017). Therefore, finding strategies to maintain the potency of the cerebral vasculature, such as increased angiogenesis, may protect against stroke.

The present study was designed to help bridge the current knowledge gap in prophylactic exercise and stroke outcome. Recognizing that many exercise regimens shown as therapeutic require long and arduous physical activity, likely not appealing to many at-risk stroke patients, we now tested whether a very short exposure to exercise was sufficient to afford neuroprotection. Here, we assessed the effects of short bouts of exercise prior to stroke induction and characterized cerebral blood flow and concomitant motor functions in vivo. We hypothesized that initiating exercise prophylactically could enhance angiogenesis and strengthen the vasculature, thereby affording neuroprotective effects against stroke.

## Methods

### Subjects

**Fig. 1:**
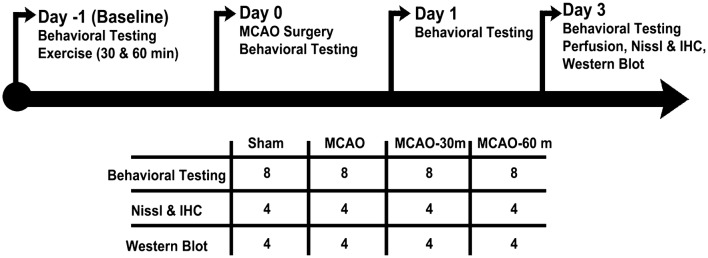
Timeline of experimental protocols and schematic showing experimental group numbers for each procedure. Procedures were performed on day − 1 (baseline), day 0, day 1, and day 3. A total of *n* = 32 animals were used. For behavioral testing, *n* = 8 animals were used for each group. For Nissl & IHC and for Western blot analyses, *n* = 4 animals were used for each group. *MCAO* middle cerebral artery occlusion, *MCAO-30m* MCAO with 30 min of exercise, *MCAO-60m* MCAO with 60 min of exercise, *IHC* immunohistochemistry

All experiments were conducted in accordance with the National Institutes of Health Guide and Use of Laboratory Animals, and were approved by the Institutional Animal Care and Use committee of the University of South Florida, Morsani College of Medicine. All animals were housed under normal conditions (20 °C, 50% relative humidity, and a 12 h light/dark cycle) and had free access to food and water. A total of 32 adult male Sprague–Dawley rats (2 months-old, 250–300 g) were used (Fig. 1).

### Surgical Procedures

Adult Sprague–Dawley rats were subjected to stroke (*n* = 24) or sham surgery (*n* = 8) and anesthetized by a mixture of 1–2% isoflurane in nitrous oxide/oxygen (69%/30%) via face mask. Body temperature was maintained at 37 ± 0.3 °C during the surgical procedures. A midline skin incision was made in the neck with subsequent exploration of the right common carotid artery (CCA), the external carotid artery, and internal carotid artery. A 4-0 monofilament nylon suture (27.0–28.0 mm) was advanced from the CCA bifurcation until it blocked the origin of the middle cerebral artery (MCA). Animals were allowed to recover from anesthesia during MCAO. After 60 min of transient MCAO, animals were reanesthetized and reperfused by withdrawal of the nylon thread. For the sham surgery, animals were anesthetized, a midline incision was made in the neck, and the right CCA was isolated. The incision was then closed and the animal was allowed to recover from anesthesia. Brain cerebral blood flow was obtained using a laser Doppler (Perimed, Periflux System 5000), with the probe placed over the right frontoparietal cortical area supplied by the MCA. Measurements were taken in terms of relative unit values during baseline, during MCAO, and after reperfusion. Physiological outcome parameters of MCAO were maintained constant across all animals throughout the entire experiment.

### Treadmill Exercise

The animals in the exercise groups were forced to run on a motorized treadmill (Lafayette Instrument; Model 80805A) for 30 and 60 min on the day before surgery. The 30 min (30m) exercise group ran at a speed of 2 m/min for the first 5 min, 5 m/min for the next 5 min, and 8 m/min for the remaining 20 min. The 60 min (60m) exercise group ran at a speed of 2 m/min for the first 5 min, 5 m/min for the next 5 min, and 8 m/min for the next 20 min, 2 m/min for the next 5 min, 5 m/min for the next 5 min, and 8 m/min for the remaining 20 min (Shimada et al. 2013). Animals in the non-exercise groups were left on the treadmill for the same period of time without running. No electrical shock or aversive stimulus was present and the treadmill was designed to prevent the animals from falling off.

### Behavioral Tests

All investigators testing the animals were blinded to the treatment condition. Each rat was subjected to a series of behavioral tests to reveal motor and neurological performances of animals before (Baseline) and after MCAO (day 0) and before perfusion (day 1 and 3). The tests included the elevated body swing test (EBST), forelimb akinesia, and paw grasp tests.

#### Elevated Body Swing Test (EBST)

EBST is a measure of asymmetrical motor behavior that does not require animal training or drug injection (Borlongan and Sanberg 1995). The rat was held, in the vertical axis, approximately 1 in from the base of its tail and then elevated to an inch above the surface on which it had been resting. The frequency and direction of the swing behavior were recorded for over 20 tail elevations. A swing was counted when the head of the rat moved more than 10° from the vertical axis to either side. Normally, intact rats display a 50% swing bias, that is, the same number of swings to the left and to the right. A 75% swing bias towards one direction was used as criterion of motor deficit (Borlongan and Sanberg 1995). The total number of swings made to the biased side was added per group and divided by the *n*, giving us the average number of swings per treatment group.

#### Forelimb Akinesia Test

During the experiment, rats from sham, non-exercise stroke, 30m exercise, and 60m exercise groups were evaluated for forelimb akinesia (Borlongan et al. 1998). Ipsilateral and contralateral forepaw strength and motility were scored by two experimentally blinded evaluators using the following forelimb akinesia scale. In a scale of 1–3, 1 = normal, 2 = impaired and 3 = severely impaired. Scores were tallied for each individual animal, then mean scores for treatment groups were used for analyses.

#### Paw Grasp Test

During the experiment, grip strength of the animals from sham, non-exercise stroke, 30m exercise, and 60m exercise groups, were evaluated for the paw grasp test. An abnormal grip is indicative of impaired neuromuscular function. In this test rats were held by their bodies against a pole (Ibrahim et al. 2009). Both ipsilateral and contralateral paw grip strength were scored by two experimentally blinded evaluators using the following grip strength scale. In a scale of 1–3, 1 = normal, 2 = impaired and 3 = severely impaired. Scores were tallied for each individual animal, then mean scores for treatment groups were used for analyses.

### Perfusion

Under deep anesthesia, rats were euthanized on day 3 for immunofluorescent and protein analysis. For immunofluorescent analysis, animals were perfused through the ascending aorta with 200 mL of cold PBS, followed by 200 mL of 4% paraformaldehyde in phosphate buffer (PB). Brains were harvested and post-fixed in the same fixative for 72 h, followed by 30% sucrose in PB until completely sunk. Series of coronal sections were cut at a thickness of 40 µm using a cryostat and stored at − 20 °C. For Western blot protein analysis, animals were perfused through the ascending aorta with 200 mL of cold PBS. Brains were harvested and coronal sections were collected at a thickness of 2 mm using a brain matrix. The ipsilateral cortex and ipsilateral striatum were collected, homogenized and stored at − 80 °C.

### Nissl Staining

Nissl staining was performed with 0.1% cresyl violet solution (Sigma-Aldrich) using a standard protocol to evaluate the peri-infarct injury of our MCAO model. From each perfused brain, six coronal sections between the anterior edge and posterior edge of the MCAO infarct area were collected and processed for Nissl staining. Every sixth coronal tissue section was chosen at random to quantify cell survival in the peri-infarct area. Brain sections were examined using a light microscope (Keyence). Neuronal survival in the peri-infarct area of the brain was quantified using a computer-assisted image analysis system (NIH Image Software, USA) and was expressed as a percentage of the ipsilateral hemisphere compared to the contralateral hemisphere.

### Immunohistochemistry Protocol

Brain and spleen sections were processed for mechanism-based immunohistochemical analyses of tissue samples focusing on inflammation. Immunofluorescence labeling was conducted on every third coronal section of the brain and every sixth coronal section of the spleen. Briefly, sections were washed three times for 10 min in 0.1 M phosphate-buffered saline (PBS). Six sections were incubated with saline sodium citrate (SSC) solution at pH 6.0 for 40 min at 80 °C for antigen retrieval purposes. Then, samples were blocked for 60 min at room temperature with 8% normal goat serum (Invitrogen, CA) in 0.1 M PBS containing 0.1% Tween 20 (PBST) (Sigma). Sections were then incubated overnight at 4 °C with corresponding primary antibody with 10% normal goat serum. The primary antibodies used for brain tissue were rabbit polyclonal angiopoietin 2 (Ang2; 1:500; Abcam; ab153934), rabbit polyclonal VEGF Receptor 2 (VEGFR2; 1:500; Novus; NB100-530), rabbit polyclonal Von Willebrand Factor (vWF; 1:500; Abcam; ab6994), and rabbit monoclonal CD34 (CD34; 1:500; Abcam; ab81289). The primary antibodies used for spleen tissue were rabbit polyclonal anti-tumor necrosis factor alpha (TNF-alpha; 1:500; ab6671) and rabbit polyclonal anti-CD45 antibody (CD45; 1:500; Abcam; ab10558). Then, sections were washed five times for 10 min in 0.1 M PBST and soaked in 5% normal goat serum in 0.1 M PBST containing corresponding secondary antibodies, goat anti-rabbit IgG-Alexa 488 (green) (1:500; Invitrogen) or goat anti-rabbit IgG-Alexa 594 (red) (1:500; Invitrogen) for 2 h. Finally, coronal sections were washed five times for 10 min in 0.1 M PBST and three times for 5 min in 0.1 M PBS, processed for 1:300 Hoechst 33258 (bisBenzimideH 33258 trihydrochloride, Sigma) for 30 min, washed in 0.1 M PBS, and cover-slipped with Fluoromount (Aqueous Mounting Medium; Sigma; F4680). Coronal sections were examined using a confocal microscope (Zeiss). Control studies included exclusion of primary antibody substituted with 5% normal goat serum in 0.1 M TBS. No immunoreactivity was observed in these controls.

### Western Blot Analysis

The ipsilateral striatum, where the stroke-induced infarct and damage occurs (Acosta et al. 2015; Ishikawa et al. 2013), was dissected and washed briefly with cold PBS to remove any blood. The tissue was cut into smaller pieces and kept on ice. Then, tissue was transferred to a homogenizer and treated with CelLytic MT mammalian lysis reagent (Sigma-Aldrich, C3228) with protease inhibitor cocktail (Sigma-Aldrich, I3786). The lysate was centrifuged at 3000×*g*, 4 °C for 15 min, and the supernatant was stored at − 80 °C until analysis. Protein samples (4–35 µg/lane) were processed on 4–14% Tris–Glycine SDS-PAGE gel and then transferred onto a nitrocellulose membrane (Bio-Rad, 162-0112) at 30 V, 4 °C for 14 h. The nitrocellulose membranes were treated with PBS containing 0.1% Tween-20 (PBST) and 3% non-fat milk (Bio-Rad, 170-6404) for 45 min at RT. Membranes were then incubated with the primary antibodies, anti-Angiopoietin 2 (Ang-2) rabbit polyclonal (1/500, Abcam Cat# ab153934), anti-VEGF Receptor 2 rabbit polyclonal (1/500, Novus Cat# NB100-530), and anti-VEGFA rabbit polyclonal (1/500, Abcam Cat# ab46154) at 4 °C for 14 h. After washing with PBST, the nitrocellulose membrane was incubated with donkey anti mouse IRDye800®CW secondary antibody (1/5000, LI-COR Biosciences Cat# 926-32212, RRID: AB_621847), or donkey anti-rabbit IRDye800®CW secondary antibody (1/5000, LI-COR Biosciences Cat# 926-32213) for 90 min at RT in the dark. Immunoreactive detection using near-infrared fluorescence was performed according to the protocol of Odyssey® Infrared Imaging System (LI-COR®).

### Statistical Analysis

The data were evaluated statistically using one-way analysis of variance (ANOVA) and subsequent post hoc Bonferroni’s test for behavior. Statistical significance was preset at *p* < 0.05. (GraphPad version 5.01). The Kolmogorov–Smirnov test was performed to assess normality and the resulting values were < 5% of the critical values.

## Results

### Quantitative Analysis of Infarct Volume

**Fig. 2:**
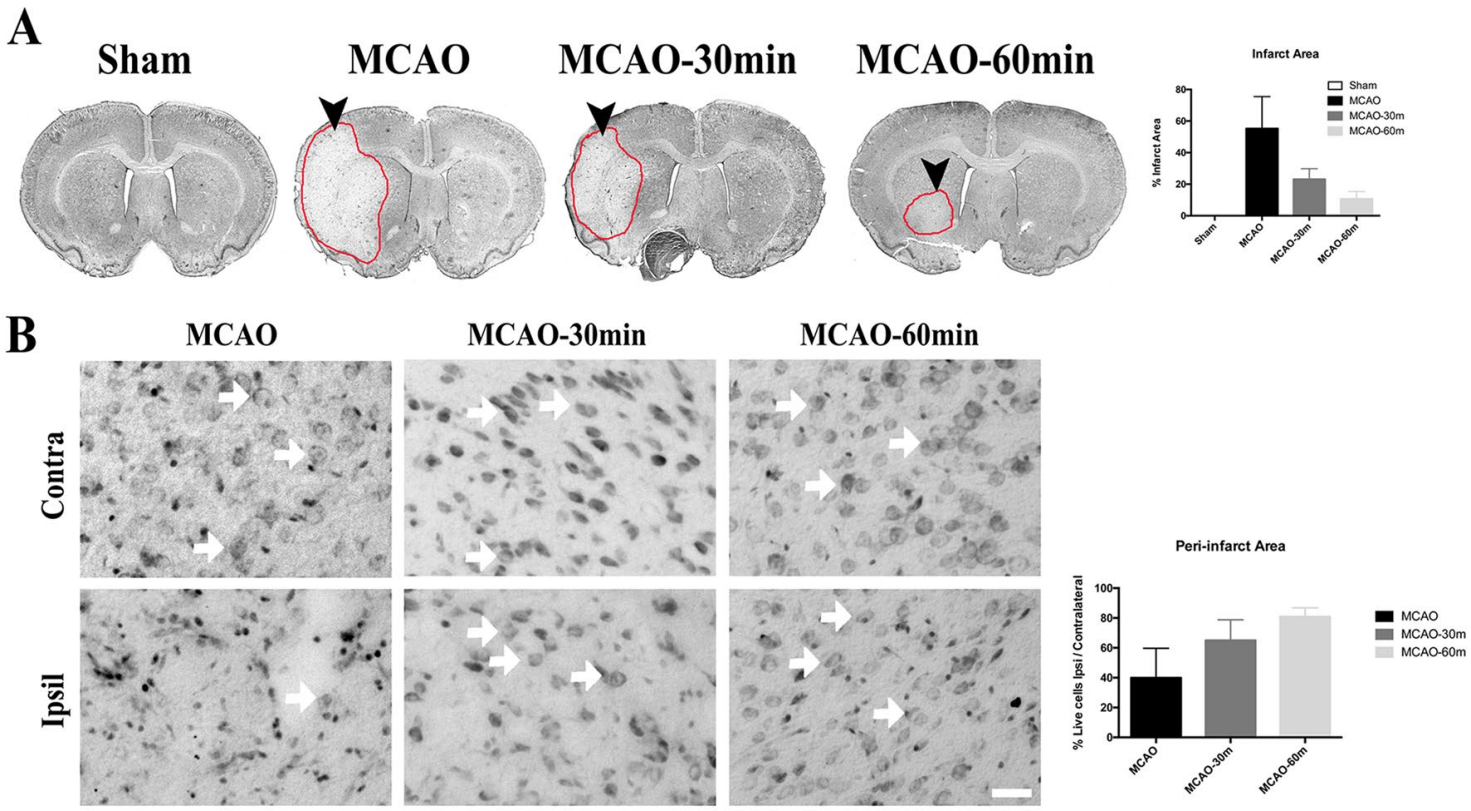
Nissl staining for sham, exercise, and non-exercise MCAO groups. **a** Quantitative analysis of the infarct volume in sham, non-exercise stroke (MCAO), and MCAO with either 30 min (MCAO-30 min) or 60 min (MCAO-60 min) of exercise groups. Coronal brain sections showing infarct area (red circle). Exercise before stroke seemed to reduce the infarct size area but no significant differences were observed between groups (*p* > 0.05). **b** Quantitative analysis of live cells in the peri-infarct area in MCAO, MCAO-30 min and MCAO-60 min groups. The chart represents the ratio between live cells in ipsilateral and contralateral regions of the brain. Live cells in the peri-infarct area were increased in the exercise groups but no significant differences were observed between groups (*p* > 0.05). Sham: (*n* = 4); MCAO: (*n* = 4); MCAO-30 min: (*n* = 4); MCAO-60 min: (*n* = 4). Error bars represent S.E.M

Animals that underwent short durations of exercise (30m and 60m exercise groups) prior to stroke induction showed reduced infarct volumes compared to non-exercised stroke animals. The exercise group of stroke rats displayed reduced infarct sizes with the 60 m group showing the smallest area of neuronal cell death (Fig. 2a). Histologically, qualitative analysis of surviving cells in the peri-infarct area showed a concomitant increase in number of live cells as the duration of exercise increased (Fig. 2b).

### Immunohistochemical Analyses of VEGFR2, Ang2, vWF, and CD34 in Peri-infarct Area

**Fig. 3:**
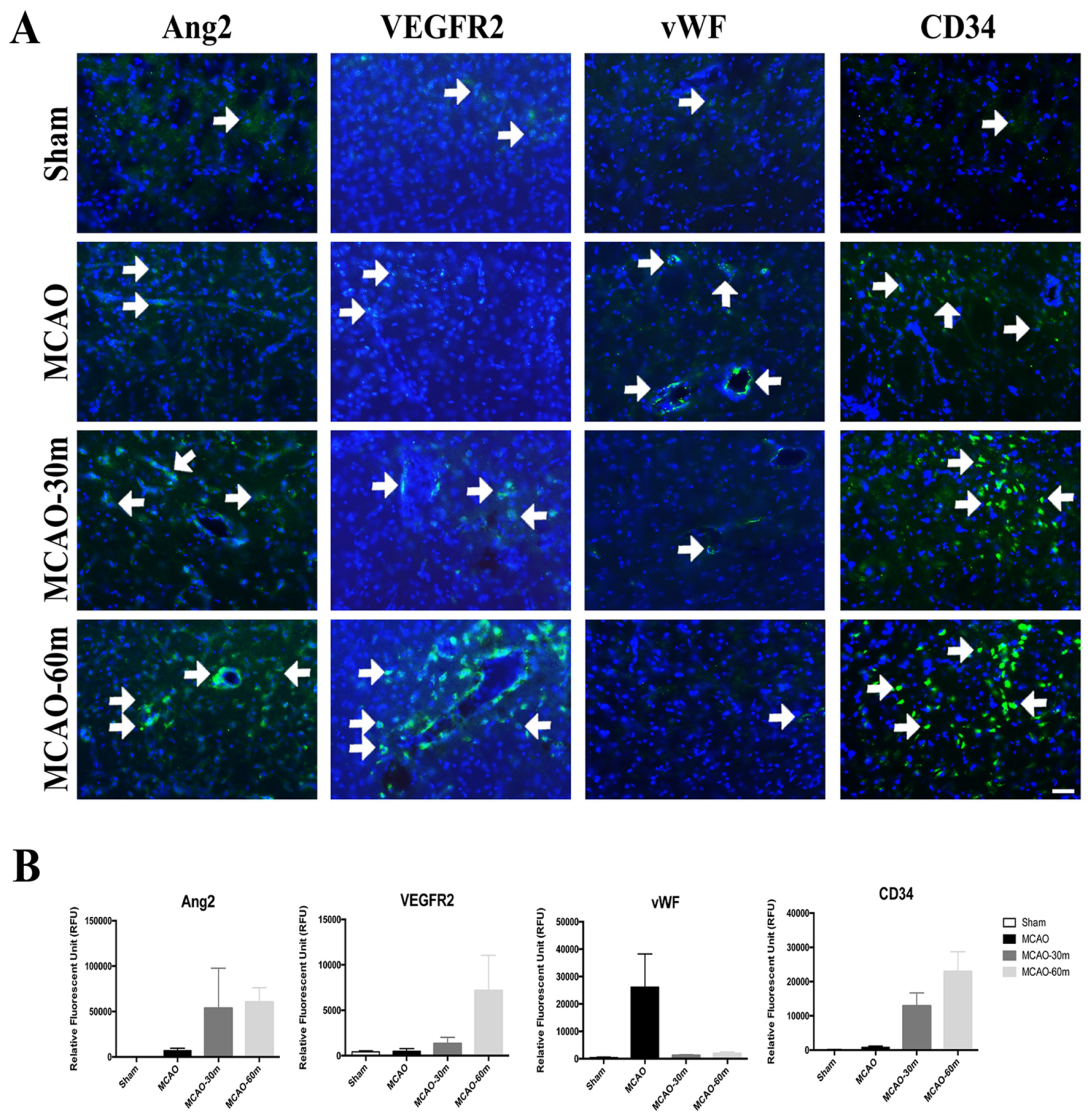
Immunohistochemical analyses of VEGFR2, Ang-2, vWF and CD34 in the peri-infarct area of the ischemic brain. **a** Representative merged images counterstained with DAPI (nuclear staining) (blue). Ang-2, VEGFR2, vWF and CD34 antibodies are represented by the green color (arrows). **b** Relative fluorescence intensity showed that Ang-2, VEGFR2, and CD34 were increased in the exercise groups. After exercise, vWF was decreased. No significant differences were observed between groups (*p* > 0.05). Sham: (*n* = 4); MCAO: (*n* = 4); MCAO-30m: (*n* = 4); MCAO-60m: (*n* = 4). Bar = 50 µm. Error bars represent S.E.M

Immunohistochemical analyses of vascular marker vascular endothelial growth factor receptor 2 (VEGFR2), angiogenesis marker angiopoietin-2 (Ang-2), and endothelial progenitor cell marker (CD34) in the peri-infarct area of the ischemic brain revealed higher quantities of each marker in the exercise group of stroke rats, with the 60m group possessing the highest quantities, while a lower number of endothelial dysfunction marker von Willebrand Factor (vWF) was observed in the peri-infarct areas of the exercise group of stroke rats relative to non-exercise stroke animals (Fig. 3).

### Evaluation of Motor Activity, Neurological Function, and Cerebral Blood Flow

**Fig. 4:**
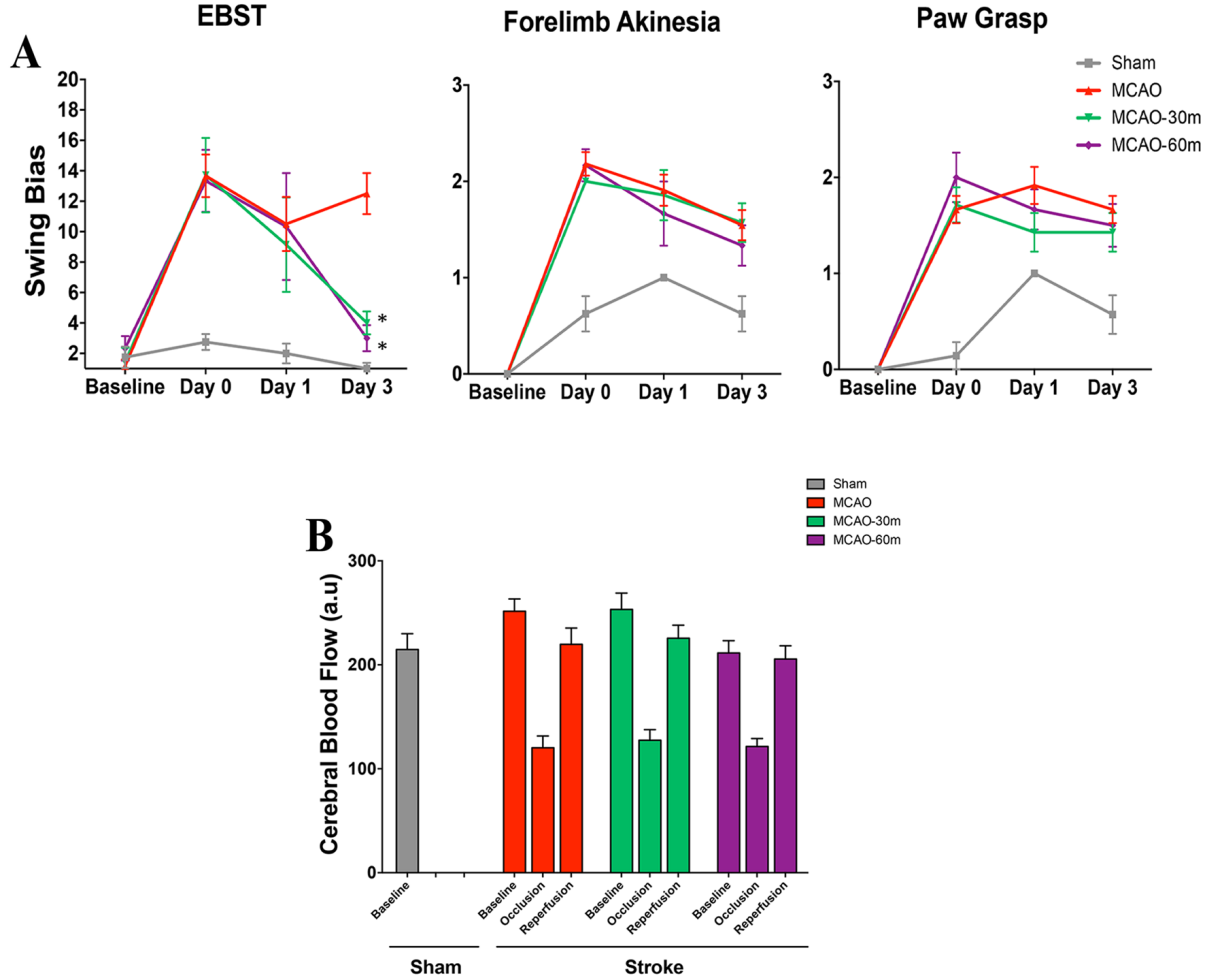
Behavioral testing and cerebral blood flow. **a** Motor activity as measured by the EBST, and neurological function as measured by forelimb akinesia and paw grasp tests. Exercise group rats showed significantly improved motor activity on day 3, as revealed by EBST. (*p < 0.05) For forelimb akinesia and paw grasp tests, no significant differences were observed between groups (*p* > 0.05). **b** Stroke animals showed a reduction in regional cerebral blood flow during the occlusion period, and an increase after reperfusion, as determined by laser Doppler. No significant differences were found between exercise and non-exercise groups (*p* > 0.05). Sham: (*n* = 8); MCAO: (*n* = 8); MCAO-30m: (*n* = 8); MCAO-60m: (*n* = 8). Error bars represent S.E.M

Behavioral tests using the EBST, forelimb akinesia, and paw grasp were conducted at baseline, day 0 (day of stroke), and at days 1 and 3 after stroke. The exercise group of stroke rats showed significant improvement in motor activity (*p* < 0.05) on day 3 compared to the non-exercise group as revealed by EBST, but forelimb akinesia and paw grasp results did not show any significant differences between the groups (Fig. 4a). Measurement of cerebral blood flow by laser Doppler at baseline (prior to MCAO), during MCAO, and during reperfusion did not show any significant difference between groups (Fig. 4b).

### Western Blot Analyses of Ang-2, VEGFR2, and VEGF in the Ipsilateral Ischemic Striatum

**Fig. 5:**
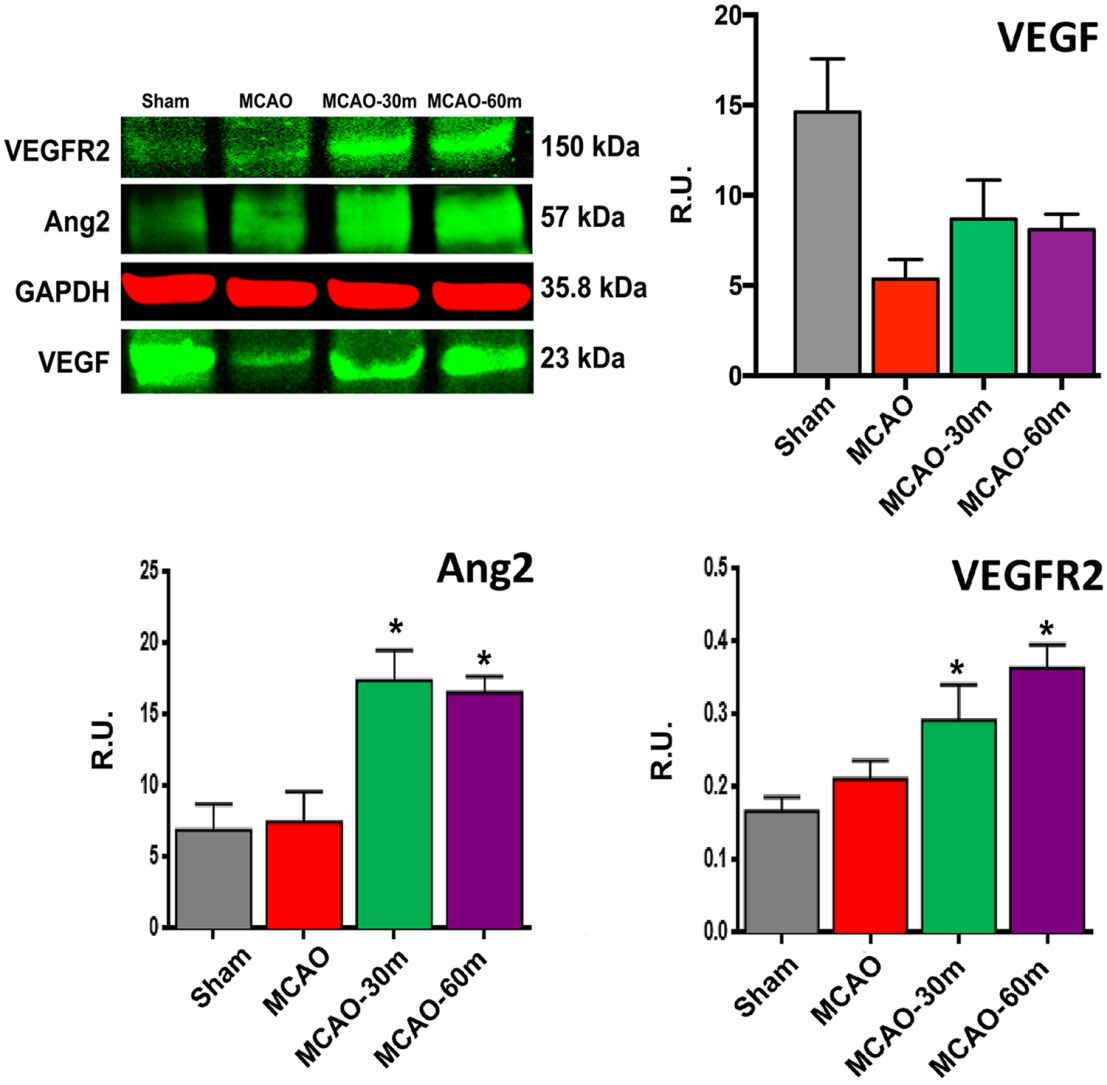
Western blot analysis of Ang2, VEGFR2, and VEGF in the ipsilateral ischemic striatum. Quantitative analysis showed a significant increase in Ang-2 and VEGFR2 expression in the ipsilateral ischemic striatum of the 30m and 60m exercise groups relative to the non-exercise group. The VEGF expression showed an increasing trend in the exercise group rats. Sham: (*n* = 4); MCAO: (*n* = 4); MCAO-30m: (*n* = 4); MCAO-60m: (*n* = 4). Data expressed as mean ± S.E.M. (*p < 0.05) were normalized to GAPDH levels. Error bars represent S.E.M

Western blot analysis of the ipsilateral ischemic striatum showed a significant increase in Ang-2 and VEGFR2 in the 30m and 60m exercise groups of stroke rats relative to the non-exercise stroke group (*p* < 0.05), while an increasing trend in VEGF was noted (Fig. 5).

### Immunohistochemical Analyses of Inflammatory Marker CD45 and TNF-Alpha in Spleen

**Fig. 6:**
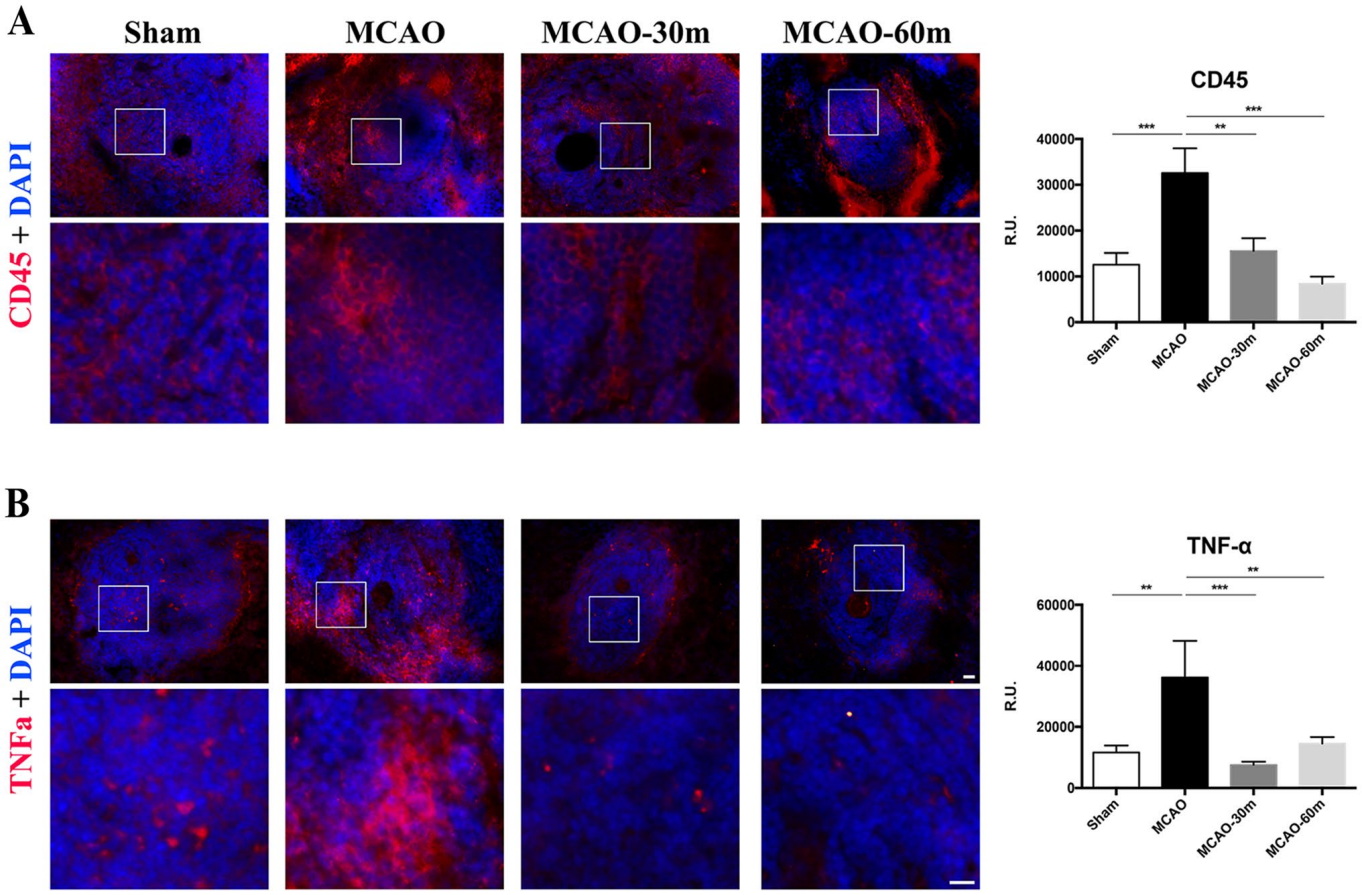
Immunohistochemical analysis of inflammatory marker CD45 (**a**) and TNF-alpha (**b**) in the spleen. Analysis of fluorescence intensities showed significantly decreased expression of inflammatory marker CD45 and TNF-alpha (red) in the spleens of exercise group rats (30m and 60m) compared with non-exercise rats. Sham: (*n* = 4); MCAO: (*n* = 4); MCAO-30m: (*n* = 4); MCAO-60m: (*n* = 4). Bar = 50 µm. Data expressed as mean ± S.E.M. (**p < 0.01, ***p < 0.001). Error bars represent S.E.M

Analysis of fluorescence intensities showed a decrease in expression of inflammatory markers CD45 and TNF-alpha in the spleens of the 30m and 60m exercise groups of stroke rats compared with non-exercise stroke rats (Fig. 6).

## Discussion

The present study demonstrated that prophylactic exercise using a short bout of a running wheel regimen was sufficient to promote neuroprotective effects against stroke. These functional benefits appear to be mediated by enhanced angiogenesis. Stroke animals subjected to exercise displayed improved motor performance and reduced histopathological deficits. In tandem, these exercise-exposed stroke animals exhibited elevated phenotypic markers of angiogenesis as evidenced by increased VEGF, VEGFR2, and Ang-2 expression in the peri-infarct area of the ischemic brain.

The current results demonstrating concurrent improvements in angiogenesis, motor activity, infarct sizes, and the quantity of surviving peri-infarct neurons parallel previous studies that highlight how treadmill exercise regimens increase angiogenesis in ischemic stroke animals (Tang et al. 2018; Gao et al. 2014), and show the crucial role of angiogenesis and the VEGF signaling pathway in rendering exercise-induced behavioral and histological benefits against ischemic stroke (Chen et al. 2019; Xie et al. 2019). Previously, 8 weeks of moderate or high intensity treadmill exercise prior to MCAO has led to improved angiogenesis, neurological function, brain infarct sizes, and blood brain barrier integrity following ischemic stroke (Rezaei et al. 2018). This is consistent with the increase in Ang-2, VEGFR2, and VEGF in the striatum, and the decrease in the infarct areas of the 30m and 60m exercise groups in the present study. Moreover, the high intensity exercise group in the prior investigation experienced the greatest decrease in infarct volume, and largest increase in markers of angiogenesis (Rezaei et al. 2018), similar to the results of the 60m exercise group in the current study. However, compared to the past report, the 30m and 60m exercise groups in the present study did not demonstrate any improvements in neurological function compared to the non-exercise stroke group. The inconsistencies in exercise-induced improvement in neurological function between the present investigation and the Rezaei et al. study may be attributed to the long-term exercise duration of the prior study, implicating that a short 30 min or 60 min exercise regimen prior to ischemic stroke is not sufficient to prevent neurological deficits post-stroke. In addition, these discrepancies may result from the different functional metrics employed to measure neurological function between the two studies. Furthermore, 3 weeks of daily 30 min exercise sessions before ischemic stroke promotes angiogenesis, increases brain-derived neurotrophic factor and midkine expression, reduces infarct volumes, neuronal apoptosis, and oxidative damage, and improves motor function but not neurological function (Otsuka et al. 2016). These past results are mimicked by the present study in which 30m and 60m exercise groups experienced increased angiogenesis, increased neuronal survival in the peri-infarct area, decreased infarct sizes, and improved motor activity but not neurological function. Based on the previous Otsuka et al. study, it is possible that these exercise-induced benefits for cerebral ischemia in the present investigation were also due to increased expression of brain-derived neurotrophic factor and midkine, and reduction of neuronal apoptosis and oxidative stress, in addition to the observed increase in angiogenesis. Interestingly, the Otsuka et al. paper suggests that 3 weeks of daily exercise prior to MCAO is not sufficient to induce neurological improvements in ischemic stroke, and reinforces the idea that a longer timeframe preconditioning exercise regimen, such as the 8 week protocol utilized in the Rezaei et al.’s investigation, is necessary to provide these enhancements in neurological function.

We demonstrated here for the first time that a single, brief 30 min or 60 min forced wheel exercise regimen prior to MCAO exerts neuroprotective effects against ischemic stroke. While our findings are consistent with previous reports (Rezaei et al. 2018; Otsuka et al. 2016; Tang et al. 2018; Gao et al. 2014; Chen et al. 2019; Xie et al. 2019) on the benefits of exercise on angiogenesis and stroke, the present investigation presents the novel idea that recent exercise prior to stroke, even as a single, abrupt instance, is sufficient to increase angiogenesis and render neuroprotection post-stroke. However, longer and repeated exercise prior to stroke may confer more benefits such as improved neurological function (Rezaei et al. 2018). Notwithstanding, the timing between the initiation of a recent bout of exercise and the induction of the ischemic event may be the most crucial aspect of exercise-induced benefits against stroke. Additionally, while other preclinical reports test the effects of the intensity (Rezaei et al. 2018) or frequency (Terashi et al. 2019) of exercise and typically use consistent 30 min sessions, the present investigation extends these findings by probing the importance of the duration of the exercise event and evaluating the difference between 30 min and 60 min exercise sessions, thus revealing the increased benefits of the 60 min exercise regimen. To this end, the present investigation also bolsters the stance that preconditioning exercise is beneficial for stroke. Furthermore, the current study is the first to examine how exercise affects inflammation in the periphery following stroke, given that stroke induces inflammation in the spleen (Xu et al. 2018; Acosta et al. 2015). Our results indicate that a single exercise regimen prior to MCAO is capable of reducing inflammation in the spleen post-stroke.

The lack of exercise-induced effects on CBF, and the absence of additional exercise parameters are some limitations of the current study. For instance, exercise did not generate any functional changes in CBF during the MCAO occlusion period relative to the non-exercise stroke groups, contrary to our expected angiogenic mechanism. It is possible that the functional correlates (i.e., increased CBF) of exercise-induced increase in angiogenesis were not captured during the selected time points post-stroke, suggesting that supra-acute, acute, and chronic CBF measurements may be needed to fully reveal the effects of exercise on stroke. Laser Doppler regional CBF may be also limited, and more sensitive blood flow techniques, such as the laser speckle approach, may detect subtle changes in blood flow. Moreover, some variables were not tested, such as a longer or shorter exercise duration, the use of both pre-stroke and post-stroke exercise regimens, or comparing forced exercise and free exercise sessions. Given the trends of the present data and the greatest benefits in the 60m exercise group, it is possible that a longer exercise bout could enhance exercise-induced neuroprotection against stroke, but it may also be ideal to construct exercise routines that are not too high in intensity (Deplanque et al. 2006, 2012; Krarup et al. 2008). Additionally, while exercise after stroke has demonstrated neuroprotective effects (Tang et al. 2018; Gao et al. 2014; Chen et al. 2019; Xie et al. 2019), it can also be detrimental depending on timing (Xing et al. 2018; Li et al. 2017; Shen et al. 2016). Future studies can evaluate the efficacy of these additional exercise-related factors, as well as probe other cell survival-enhancing pathways, in order to advance exercise-based therapies for stroke.

The current data in the context of previous stroke and exercise studies indicate possible guidelines for using exercise to protect against stroke. Weak to moderate exercise prior to a stroke in humans reduces stroke severity, with longer durations of exercise associated with increased neuroprotection (Deplanque et al. 2006, 2012). Additionally, with current U.S. guidelines for stroke prevention recommending a minimum of 40 min of moderate aerobic exercise, 3–4 days per week (Howard and McDonnell 2015); and with greater neuroprotective effects observed in the 60 min exercise group in the present study, it is possible that longer durations of exercise such as 40–60 min moderate intensity sessions, are more effective for reducing stroke severity than briefer 20–30 min exercise regimens. However, optimizing and translating exercise durations from animal models to humans warrants additional research. Of note, frequent exercise appears to be an ideal prophylactic regimen for reducing stroke-induced damage. For instance, over three instances of exercise per week before stroke is most effective for mitigating the neuronal apoptosis that accompanies stroke in rats (Terashi et al. 2019), and long-term, consistent exercise prior to experimental cerebral ischemia promotes post-stroke neuroprotection and preservation of neurological function (Rezaei et al. 2018). Furthermore, since the current investigation demonstrated the importance of the timing between the initiation of a recent bout of exercise and the induction of the ischemic event in exercise-induced benefits against stroke, it is speculated that daily exercise will be imperative to not only prevent a stroke (Howard and McDonnell 2015), but to maintain these neuroprotective advantages in case an ischemic stroke develops.

Altogether, the present results demonstrated that exercise can be used as a prophylactic treatment to reduce the motor and histopathological deficits that accompany an ischemic stroke. Indeed, a single, brief bout of exercise before stroke was sufficient to induce these neuroprotective benefits, likely via the observed increase in angiogenesis, sans the CBF effects. In the clinic, we envision that even a modest exercise may minimize brain damage in the event of an ischemic stroke.
